# Syndemic, mental health and living with dependent persons in Latin America and Spain: a study with a gender perspective

**DOI:** 10.1186/s13690-024-01480-5

**Published:** 2025-01-26

**Authors:** Natalia López-Contreras, Tomás López-Jiménez, Laura Medina-Perucha, Brenda Biaani León-Gómez, Alessandra Queiroga Gonçalves, Olivia Janett Horna-Campos, Maria Sol Anigstein, Jakeline Ribeiro Barbosa, Mariana Pastorello Verotti, Olga Bardales-Mendoza, Karen M. Arteaga-Contreras, Anna Berenguera, Andrés Peralta, Constanza Jacques-Aviñó

**Affiliations:** 1 Vicerrectoría Investigación y Postgrado, Universidad de La Frontera, Temuco, Chile; 2 Fundació Institut Universitari per a la Recerca a l’Atenció Primària de Salut Jordi Gol i Gurina (IDIAPJGol), Gran Via de les Corts Catalanes, 587 attic., Barcelona, 08007 Spain; 3 Universitat Autònoma de Barcelona, Bellaterra, Barcelona, Cerdanyola del Vallès Spain; 4 Red de Investigación en Cronicidad, Atención Primaria y Promoción de la Salud (RICAPPS), Barcelona, España; 5 Unitat de Suport a la Recerca Metropolitana Nord, Fundació Institut Universitari per a la recerca a l’Atenció Primària de Salut Jordi Gol i Gurina (IDIAPJGol), Mataró, Spain; 6 Unitat de Suport a la Recerca Terres de l’Ebre, Fundació Institut Universitari per a la recerca a l’Atenció Primària de Salut Jordi Gol i Gurina (IDIAPJGol), Tortosa, Tarragona, 43500 Spain; 7 Escuela de Salud Pública “Salvador Allende”, Facultad de Medicina, Universidad de Chile, Santiago, Chile; 8 Departamento de Antropología, Universidad de Chile, Santiago de Chile, Chile; 9 Center for Epidemiology and Health Surveillance, Oswaldo Cruz Foundation, Brasília, Brazil; 10 Universidad Peruana Cayetano Heredia, Lima, Peru; 11 Hospital Psiquiátrico Fray Bernardino Álvarez de los Servicios de Atención Psiquiátrica, Cuidad de México, México; 12 Departament d’Infermeria, Universitat de Girona, Girona, Spain; 13 Public Health Institute, Pontificia Universidad Católica del Ecuador (PUCE), Quito, Ecuador

**Keywords:** Lockdown, Mental health, Health inequities, COVID-19, Gender perspective

## Abstract

**Objective:**

To analyze the sociostructural determinants associated with mental health problems during the lockdown period among populations residing in Brazil, Chile, Ecuador, Mexico, Peru, and Spain who lived with minors or dependents, approached from a gender perspective.

**Methods:**

A cross-sectional study was conducted in six participating countries via an adapted, self-managed online survey. People living with minors and/or dependents were selected. Multivariate logistic regression models were estimated to assess the associations between sociostructural variables and mental health problems (anxiety (GAD-7) and/or depression (PHQ-9)). The analyses were stratified by sex and country.

**Results:**

Out of a total of 39,006 people, 18,040 reported living with minors and/or dependents (73% women). In all countries, women reported worse mental health, with Spain having a lower prevalence. The risks of mental health problems in women in most countries are associated with poor housing conditions and performing care work. University education was associated with a protective factor. For men, risks were related to being younger, worsening working conditions and concerns about living together at home.

**Conclusions:**

Women in Latin America who lived with dependents had worse outcomes than those in Spain did. It is necessary to develop intersectoral and social determinants strategies to prevent, protect and support the mental health of those who live with dependents and minors.

## Resumen

**Objetivo:**

Analizar los determinantes socioestructurales asociados a problemas de salud mental durante la primera ola del confinamiento en la población residente en Brasil, Chile, Ecuador, México, Perú y España que convivieron con personas dependientes desde una perspectiva de género.

**Métodos:**

Se realizó un estudio transversal mediante una encuesta adaptada y en línea autogestionada en los seis países participantes. Se seleccionaron a personas que convivían con personas menores y/o dependientes. Se calcularon modelos de regresión logística multivariante para evaluar la asociación entre las variables socioestructurales con problemas de salud mental (ansiedad (GAD-7) y/o depresión (PHQ-9)). Los análisis se estratificaron por sexo y país.

**Resultados:**

De un total de 39.006 personas, 18.040 personas declararon convivir con personas menores y/o dependientes (73% mujeres). En todos los países las mujeres reportaron peor salud mental, siendo España con una prevalencia más baja. Los riesgos de problemas de salud mental en las mujeres de la mayoría de los países se asociaron a las malas condiciones de vivienda y realizar trabajos de cuidados. El nivel educativo universitario se asoció a un factor protector. Para los hombres, los riesgos estuvieron relacionados con tener menor edad, empeoramiento en la condiciones laborales y preocupaciones sobre la convivencia en el hogar.

**Conclusiones:**

Las mujeres de Latinoamérica que convivieron con personas dependientes presentaron peores resultados que las de España. Es necesario desarrollar estrategias intersectoriales y desde los determinantes sociales para prevenir proteger y respaldar la salud mental de quienes conviven con personas dependientes y menores.

**Table:** 

Text box 1. Contributions to the literature
• Most of the available evidence on confinement and its impact on the mental and physical health of the population comes from Europe.
• Performing unpaid care work, an important factor of gender inequality, has negative consequences for mental health.
• There is a higher risk of mental health problems in women living with dependent persons and associated with socio-structural determinants in most of the Latin American countries analyzed. The best results were obtained in Spain.
• It is crucial to develop strategies that support caregivers, promoting support networks and an equitable distribution of responsibilities, which are fundamental to sustain life in society.

## Background

People from the most socioeconomically disadvantaged backgrounds were disproportionately affected by the syndemic impact of the Coronavirus Disease 2019 (COVID-19) [1, 2]. While social distancing measures varied from country to country, lockdown policies exacerbated existing inequities in the incidence and mortality of the disease, and further entrenched existing social inequalities [1]. In this context, research conducted during lockdowns indicates that women bear a disproportionate responsibility for caregiving tasks [3]. The term ‘unpaid care work’ (UCW) encompasses activities conducted both inside and outside the home that sustain the physical and emotional well-being of those receiving care [4]. Feminist economics highlights the importance of recognizing the economic value of UCW in terms of social and family reproduction [5].

Although UCW are not inherently male or female, social expectations shaped by the prevailing capitalist, patriarchal, and colonial systems tend to ascribe this responsibility to women [6]. Viewing gender as a ender as a historical category enables the identification of power relationships that generate inequalities through segregation and discrimination against a section of the population [6]. It can be argued that the UCW is fundamental for the survival of minors, as they provide support for the “productive” work that is mainly performed by men. However, this work is undervalued in prevailing economic and social models [5]. The sexual division of labor has a detrimental impact on women’s mental health, particularly under conditions of excessive workloads and stress [7].

Prior research has shown that living with dependents or minors may intensify the mental and physical tolls associated with syndemic, influencing both mental health [8] and self-perceived health [9]. From a feminist perspective, psychological development should emphasize connection and relationships, rather than models that promote self-sufficiency and independence as hallmarks of growth [3]. In this way, connections become the backbone of social reproduction, especially to cushion the precarious lives of many women who are involved in care, whether formal or informal [10].

In the Latin American context, characterized by high levels of socioeconomic inequality, ongoing social crises and a lack of equitable access to job opportunities and basic services, the care crisis has become even more acute [11]. The prevalence of neoliberal policies in several Latin American countries has exacerbated social class disparities due to the absence of guaranteed social entitlements and gender-based inequalities [12]. Over recent decades, the pursuit of reduced state involvement and greater economic liberalization, these policies have resulted in the weakening of social protection systems and a decline in the provision of public services [13]. Consequently, only a few Latin American countries included in this study have care systems in place. In most of these countries, there is a lack of consistency in the measures for maternity and dependent care leave [14–16], with long-term care services also being scarce [17–19] (Table 1). In Spain, the “Dependency Law” (approved in 2006) provides services and compensation for those requiring care at home. However, its implementation has been [20]. Conversely, during the syndemic period, educational establishments were forced to close for nearly two years, as was the case in several Latin American countries [21]. Consequently, the lack of political support and government initiatives has intensified social demands and multiple responsibilities, thereby exacerbating the burden of UCW on women [11].

**Table 1:** Regulations linked to policies, care services and regulation of time for care in Brazil, Chile, Ecuador, Peru and Mexico

	Brazil	Chile	Ecuador	Perú	México
**Laws on integrated care systems**	Decree 11.460 (2023) creating the Interministerial Working Group with the objective of elaborating the proposal for the National Care Policy and the proposal for the National Care Plan in Brazil.	Law 20.379, enacted in September 2009, creates the Intersectoral System of Social Protection and institutionalizes the Subsystem of Integral Protection for Children ‘Chile Crece Contigo’, which accompanies the development process of children who receive care in the public health system.	Since 2017, Ecuador has had the draft Organic Law of the National Integrated Care System in the pipeline.	In July 2022, the President of Peru submitted a project to Parliament, which recognizes the right to care for dependent persons and creates the National Care System. Wawa Wasi National Program (1993): designed to promote the optimal development of impoverished children aged 6-48 months, promote and develop an appropriate parenting culture; and contribute to the personal development of women and raise their quality of life.	In Mexico, since 2020 there has been a draft general law that creates the National Care System with the aim of articulating existing programmed and actions.
**Maternity leave**	18 weeks and +	18 weeks and +	Less than 14 weeks	14 -18 weeks	Less than 14 weeks
**Breastfeeding**	1 hour per day for 6 months	At least 1 hour for two years	2 hours the first year	1 hour per 1 year of breastfeeding	1 hour per 1 year of breastfeeding
**Paternity leave**	5 to 20 days in total	5 days	10 to 25 days	10 to 30 days	5 days
**Leave for the care of elderly and dependent adults**	Leave for family health treatment: Leave to which the public servant is entitled due to illness of a direct family member or dependent who lives at his/her expense and is included in the functional liquidation, whose care does not allow him/her to carry out the activities of the position.	Payment Program for Caregivers of Persons with Disabilities, which establishes the payment of remuneration for those who work as unpaid caregivers of persons with severe dependency or disability.	Bono Joaquín Gallegos Lara is aimed at people with severe, very severe and complete physical, intellectual and psychosocial disabilities; people with catastrophic, rare and orphan diseases; and children and adolescents under 18 years of age living with HIV-AIDS in poverty and extreme poverty.	Workers may have up to seven days per year to care for family members with health problems.	By the year 2021, there will be no more
**Childcare services in the workplace**	Obligation in the case of companies with more than 30 female workers over 16 years of age with children under 5 years of age.	Companies with more than 20 female employees must provide on-site childcare, in conjunction with other employers, or pay for the cost of childcare.	Companies with more than fifty workers and/or workers with children under seven years of age must have a nursery attached to the workplace.	Implementation of Day Care Services: where more than 50 women of childbearing age work and/or provide services and/or where workers require day care services for their children, in a number of no less than 16 children.	The General Law on the Provision of Services for the Attention, Care and Comprehensive Development of Children, reformed in 2018, guarantees children's access to such services under equal and quality conditions, up to the age of four.

However, despite the passage of time since the crisis, the syndemic has significantly impacted social and psychological well-being and has highlighted concerns about potential environmental, socioeconomic and other emergencies. In light of this, we propose an investigation into the impact of the care crisis on adults who have lived with individuals requiring care, with particular attention to the sociopolitical context of the region. This is especially important given the potential implications for the present and future. Accordingly, the objective of this study was to analyze the sociostructural determinants associated with mental health problems during the lockdown period among populations residing in Brazil, Chile, Ecuador, Mexico, Peru, and Spain who lived with minors or dependents, approached from a gender perspective.

## Methods

### Study design and data source

A descriptive, population-based, cross-sectional study was conducted via a self-administered online survey targeting individuals aged 18 years and older residing in Brazil, Chile, Ecuador, Mexico, Peru and Spain. The data were collected in 2020, during the period of lockdown associated with the initial phase of the pandemic.

The questionnaire was adapted to align with the specific characteristics of each country. A pilot study was conducted in order to adapt the questions to the sociodemographic diversity of the populations under investigation. At the beginning of the survey, the objective of the study was explained, and participants were asked to review and sign an informed consent document. In Spain, the REDCap platform, which is a secure web-based tool designed to facilitate data capture in research studies, was employed. The platform offers an intuitive interface for validated data collection, an audit trail to track data manipulation and export, automated procedures for seamless data downloads to common statistical packages, and integration and interoperability procedures with external sources [22, 23]. In the case of Latin America, the survey data were collected and managed via SurveyMonkey^®^, an electronic data capture tool hosted by Institut de Recerca en Atenció Primària Jordi Gol i Gurina (IDIAPJGol).

This study was approved by the Research Ethics Committee of the IDIAPJGol with approval number 20/063-PCV. The participants provided online written consent. All methods were carried out in accordance with the Declaration of Helsinki.

### Sampling

Each research team was responsible for data collection via online platforms in their respective countries, using social networks for dissemination. Convenience and snowball sampling techniques were employed. To be included in the study, participants had to be at least 18 years of age and reside in the country in question.

### Definition of mental health

The main study variable was mental health, with underlying emotional distress serving as a key explanatory factor [24]. The scale was constructed based on the subject’s scores on the Generalized Anxiety Disorder Scale (GAD-7), which indicates whether the subject presents with “moderate to severe” anxiety, and/or the score on the Patient Health Questionnaire (PHQ-9), which indicates whether the subject presents with “moderate/severe” depressive symptoms. Anxiety was defined as persistent worry and anticipatory responses to future threats. Depression was defined as marked feelings of sadness, emptiness, or irritability [25]. Furthermore, the two scales have been validated at the international level and in each country that participated in the study, with high levels of validity and reliability [26, 27]. Consequently, the variable was categorized as follows: people with or without mental health problems.

### Covariates

The variables related to social factors were gender (female‒male), educational level (non-university‒university), age (18 to 34 years‒35 to 64 years‒65 years and older), employment status prior to the onset of the syndemic (employed‒unemployed), and change in employment status during the syndemic (no change/improved‒worsened). The variables related to housing were tenure (owner-occupier or tenant), perception of adequate housing (good or bad), performance of housework (shared with others‒primarily performed by oneself), concern about living with household members (not at all or moderately/greatly concerned), and concern about schooling (no children or not at all/slightly, moderately or greatly concerned).

### Statistical analysis

A descriptive analysis of the variables was conducted, and the differences between individuals with and without mental health problems were compared using the chi-squared test. Multivariate logistic regression models were developed to assess the associations between the independent variables and mental health. Adjusted odds ratios (aORs) with 95% confidence intervals were calculated to ascertain the strength of the associations between the independent variables and mental health. All analyses were stratified by sex and country of residence. The level of statistical significance was set at 0.05. All analyses were conducted using the Stata 24.0 statistical software package (StataCorp LLC., College Station, Texas, USA).

## Results

Data from the 39,006 individuals who participated in the survey were analyzed, focusing specifically on the 18,040 respondents who reported living with minors and/or dependents. Table 2 presents a description of the sociodemographic characteristics of the study population. The countries represented, in descending order of prevalence, were Brazil (38.5%), Mexico (25.5%), Chile (15.9%), Spain (7.8%), Ecuador (8.4%) and Peru (3.9%). The majority of respondents were women, with the highest proportion in Brazil (79.7%), Spain (73.4%), Mexico (71.4%), Peru (70.3%), Ecuador (68.9%) and Chile (63.8%). The most represented age group was 35–64 years was 35–64 years old, with the highest proportion in Spain (86.4%), Brazil (70.3%), Chile (69%). The majority of respondents were women, with the highest proportions observed in Brazil (79.7%), Spain (73.4%), Mexico (71.4%), Peru (53.4%), Ecuador (51.4%) and Mexico (49.6%). The majority of respondents had completed university studies in all countries, with the highest proportion observed in Ecuador (84.2%), Spain (73.2%), Brazil (72.5%), Peru (68.8%), Chile (67.4%) and Mexico (65.2%). In terms of mental health problems, the prevalence of mental health problems shows gender differences across the studied countries. Women consistently reported higher rates than men, with the highest proportions observed in Chile (66.8% of women and 51% of men), followed by Ecuador (54.1% of women and 43.7% of men), Mexico (53.2% of women and 37.3% of men), Brazil (49.3% of women and 36.4% of men), Peru (50.4% of women and 36% of men), and Spain (40.4% of women and 25.1% of men).

**Table 2:** Sociodemographic characteristics and variables related to social and material factors of participants by sex in Brazil, Chile, Ecuador, Mexico, Peru and Spain in the first wave of the COVID-19

		**Brazil**										**Chile**									
		*n*=6946										*n*= 2873									
		Men					Women					Men					Women				
		No MH problem		With MH problem			No MH problem		With MH problem			No MH problem		With MH problem			No MH problem		With MH problem		
		n	**%**	n	**%**	*p*	n	**%**	n	**%**	*p*	n	**%**	n	**%**	*p*	n	**%**	n	**%**	*p*
Total		898	63,6	513	36,4		2805	50,7	2730	49,3	*	510	49,0	530	51		608	33,2	1225	66,8	*
**Anxiety**	No anxiety	898	100	111	21,6		2805	100	501	18,4		510	100	64	12,1		608	100	96	7,8	
	With anxiety	0	0	402	78,4		0	0	2229	81,7		0	0	466	87,9		0	0	1129	92,2	
**Depression**	No depression	898	100	118	23,0		2805	100	528	19,4		510	100	205	38,7		608	100	431	35,2	
	With depression	0	0	395	77,0		0	0	2202	80,7		0	0	325	61,3		0	0	794	64,8	
**Educational level**	Basic o primary studies	173	19,4	134	26,3	***	449	16,1	653	24,2	***	61	12,0	105	20	***	70	11,6	208	17,1	**
	Technical studies	68	7,6	57	11,2		143	5,1	214	7,9		83	16,4	99	18,9		89	14,7	215	17,7	
	University studies	649	72,9	318	62,5		2190	78,7	1833	67,9		363	71,6	321	61,1		445	73,7	791	65,2	
**Age**	18-34	157	17,5	209	40,7	***	434	15,5	857	31,4	***	88	17,3	166	31,3	***	88	15,9	399	32,6	***
	35-64	632	70,4	288	56,2		2170	77,4	1792	65,6		373	73,1	360	67,9		485	79,8	789	64,4	
	≥65	109	12,1	16	3,1		201	7,2	81	2,9		49	9,6	4	0,8		26	4,3	37	3,0	
**Pre-pandemic employment status**	Employed	521	72,7	282	67,8		1326	60,6	1255	58,5		438	88,8	452	90,6		478	81,2	902	77,4	
	Unemployed	196	27,3	134	32,2		863	39,4	891	41,5		55	11,2	47	9,4		111	18,9	263	22,6	
**Change in employment status during the pandemic**	No change/improvement	465	51,9	157	30,8	***	1495	54,0	1039	38,5	***	221	43,3	172	32,5	***	314	51,8	471	38,6	***
	Worsened	430	48,0	353	69,2		1273	45,9	1660	61,5		289	56,7	357	67,5		292	48,1	750	61,4	
**Housing tenure**	Own home	685	76,6	316	61,9	***	2137	76,5	1811	66,5	***	342	67,3	303	57,3	**	414	68,6	662	54,2	***
	Lease or rent	173	19,4	147	28,8		504	18,1	666	24,4		121	23,8	159	30,1		137	22,5	367	30,1	
	Living in someone else´s home	36	4,0	47	9,2		152	5,4	247	9,1		45	8,9	67	12,7		54	8,9	192	15,7	
**Perception of adequate housing**	Suitable for confinement	550	61,4	228	44,6	***	1783	63,7	1307	47,9	***	344	67,5	271	51,2	***	468	76,9	718	58,8	***
	Not suitable for confinement	346	38,6	283	55,4		1017	36,3	1418	52,0		166	32,6	258	48,8		140	23,0	503	41,2	
**Household work**	Other persons/equally	763	85,1	396	77,3	***	1305	46,7	1104	40,6	***	455	89,4	431	81,3	***	318	52,4	587	48,0	
	Mostly by myself	134	14,9	116	22,7		1492	53,3	1618	59,4		54	10,6	99	18,7		289	47,6	636	52,0	
**Concern about living with household members**	Nothing or little	314	35,0	92	18	***	1025	36,7	654	23,9	***	238	46,9	152	28,7	***	356	58,7	448	36,7	***
	Moderate, quite a bit or a lot	583	65	419	82		1772	63,4	2074	76,0		270	53,2	378	71,3		251	41,4	774	63,3	
**Concern about schooling**	Nothing or little	86	9,7	42	8,3		400	14,4	339	12,6		132	26,4	113	21,9		215	36,3	355	29,3	
	No minors	428	48,4	235	46,3	**	1102	39,8	1006	37,4	**	115	23,0	148	28,6		132	22,3	299	24,7	**
	Moderate, quite a bit or a lot	371	41,9	231	45,5		1268	45,8	1345	50,0		254	50,7	257	49,6		245	41,4	556	45,9	

		**Ecuador**										**México**									
		*n*=1507										*n*=4595									
		Men					Women					Hombres *N*=1313					Mujeres *N*=3282				
		No MH problem		With MH problem			No MH problem		With MH problem			No MH problem		With MH problem			No MH problem		With MH problem		
		n	**%**	n	**%**	*p*	n	**%**	n	**%**	*p*	n	**%**	n	**%**	*p*	n	**%**	n	**%**	*p*
Total		264	56,3	205	43,7		476	45,9	562	54,1	*	823	62,7	490	37,3		1537	46,8	1745	53,2	*
**Anxiety**	No anxiety	264	100	41	20,0		476	100	80	14,2		823	100	102	20,8		1537	100	339	19,4	
	With anxiety	0	0	164	80,0		0	0	482	85,8		0	0	388	79,2		0	0	1406	80,6	
**Depression**	No depression	264	100	57	27,8		476	100	135	24,0		823	100	118	24,1		1537	100	376	21,6	
	With depression	0	0	148	72,2		0	0	427	75,9		0	0	372	75,9		0	0	1369	78,5	
**Educational level**	Basic o primary studies	25	9,5	24	11,9		43	9,1	84	15,1	**	49	5,5	25	4,9	**	59	2,1	79	2,9	**
	Technical studies	12	4,6	6	2,9		17	3,6	25	4,5		197	22,1	159	31,2		439	15,8	585	21,7	
	University studies	225	85,9	172	85,2		412	87,3	447	80,4		576	64,7	305	59,9		1034	37,2	1072	39,7	
**Age**	18-34	86	32,6	82	40,0		227	47,7	318	56,6	**	326	36,3	267	52,0	***	640	22,8	981	35,9	***
	35-64	170	64,4	118	57,6		245	51,5	241	42,9		455	50,7	217	42,3		866	30,9	742	27,2	
	≥65	8	3,03	5	2,4		4	0,8	3	0,5		42	4,7	6	1,2		31	1,1	22	0,8	
**Pre-pandemic employment status**	Employed	222	85,1	169	82,8		371	78,3	387	69,4	**	586	81,7	322	77,4	**	949	43,4	1033	48,1	
	Unemployed	39	14,9	35	17,2		103	21,7	171	30,7		237	22,7	186	33,0		588	26,9	712	33,2	
**Change in employment status during the pandemic**	No change/improvement	96	36,6	46	22,6	**	157	33,1	124	22,2	***	451	50,4	181	35,5	***	824	29,8	666	24,7	***
	Worsened	166	63,4	158	77,4		317	66,9	434	77,8		372	41,6	309	60,6		713	25,8	1079	40,0	
**Housing tenure**	Own home	193	73,1	132	64,7		344	72,6	360	64,3	**	577	64,5	313	61,4		1050	37,6	1056	38,8	***
	Lease or rent	49	18,6	52	25,5		86	18,1	133	23,8		129	14,4	91	17,8		231	8,3	304	11,2	
	Living in someone else´s home	22	8,3	20	9,8		44	9,3	67	11,9		117	13,1	86	16,9		256	9,2	385	14,1	
**Perception of adequate housing**	Suitable for confinement	164	62,1	113	55,1		323	68,0	306	54,6	***	404	49,1	194	39,6	**	741	48,2	648	37,1	***
	Not suitable for confinement	100	37,9	92	44,9		152	32,0	255	45,5		419	50,9	296	60,4		796	51,8	1097	62,9	
**Household work**	Other persons/equally	237	90,5	176	86,3		326	68,6	329	58,5	**	734	89,2	420	85,7		1048	68,2	1045	59,9	***
	Mostly by myself	25	9,5	28	13,7		149	31,4	233	41,5		89	10,8	70	14,3		489	31,8	700	40,1	
**Concern about living with household members**	Nothing or little	131	49,6	64	31,4	***	253	53,2	176	31,4	***	435	52,9	160	32,7	***	855	55,6	675	38,7	***
	Moderate, quite a bit or a lot	133	50,4	140	68,6		223	46,9	385	68,6		388	47,1	330	67,3		682	44,4	1070	61,3	
**Concern about schooling**	Nothing or little	35	13,3	15	7,3		54	11,3	40	7,1		165	20,5	94	18,2		339	22,1	326	18,7	
	No minors	58	21,9	58	28,3		130	27,3	153	27,3		180	21,9	135	27,5		398	25,9	423	24,2	**
	Moderate, quite a bit or a lot	171	64,8	132	64,4		292	61,3	368	65,6		478	50,1	261	53,3		800	52,0	996	57,1	

		**Perú**										**España**									
		*n*= 711										*n*= 1408									
		Hombres *N*=211					Mujeres *N*=500					Hombres *N*=374					Mujeres *N*=1034				
		No MH problem		With MH problem			No MH problem		With MH problem			No MH problem		With MH problem			No MH problem			With MH problem	
		n	**%**	n	**%**	*p*	n	**%**	n	**%**	*p*	n	**%**	n	**%**	*p*	n	**%**	n	**%**	*p*
Total		135	64,0	76	36,0		248	49,6	252	50,4	*	280	74,9	94	25,1		616	59,6	418	40,4	*
**Anxiety**	No anxiety	135	100	16	21,1		248	100	51	20,2		280	100	13	13,8		616	100	53	12,7	
	With anxiety	0	0	60	78,9		0	0	201	79,8		0	0	81	86,2		0	0	365	87,3	
**Depression**	No depression	135	100	25	32,9		248	100	63	25,0		280	100	36	38,3		616	100	149	35,6	
	With depression	0	0	51	67,1		0	0	189	75,0		0	0	58	61,7		0	0	269	64,4	
**Educational level**	Basic o primary studies	12	8,8	14	18,7		28	11,3	53	21,2	**	52	18,6	22	23,4		64	10,4	88	21,1	***
	Technical studies	17	12,5	12	16,0		37	15,0	47	18,8		33	11,8	11	11,7		57	9,3	50	12,0	
	University studies	107	78,7	49	65,3		182	73,7	150	60,0		195	69,6	61	64,9		494	80,3	280	67,0	
**Age**	18-34	33	24,4	38	50,0	**	101	40,7	142	56,3	**	25	8,9	15	16,0		62	10,1	69	16,5	**
	35-64	99	73,3	36	47,4		140	56,5	105	41,7		245	87,5	78	83,0		546	88,6	347	83,0	
	≥65	3	2,2	2	2,6		7	2,8	5	2,0		10	3,6	1	1,1		8	1,3	2	0,5	
**Pre-pandemic employment status**	Employed	121	91,0	57	75,0	**	180	73,8	160	63,7	**	229	81,8	79	84,0		518	84,2	323	77,3	**
	Unemployed	12	9,0	19	25,0		64	26,2	91	36,3		51	18,2	15	16,0		97	15,8	95	22,7	
**Change in employment status during the pandemic**	No change/improvement	71	55,0	24	35,8	**	109	51,4	85	39,4	**	165	58,9	39	41,5	**	365	59,3	197	47,1	***
	Worsened	58	45,0	43	64,2		103	48,6	131	60,6		115	41,1	55	58,5		251	40,7	221	52,9	
**Housing tenure**	Own home	95	70,4	39	52,0	**	164	66,1	149	59,4		208	74,8	68	72,3		475	77,2	328	79,0	
	Lease or rent	28	20,7	24	32,0		44	17,7	65	25,9		60	21,6	23	24,5		124	20,2	77	18,6	
	Living in someone else´s home	12	8,9	12	16,0		40	16,1	37	14,7		10	3,6	3	3,2		16	2,6	10	2,4	
**Perception of adequate housing**	Suitable for confinement	62	45,9	25	32,9		112	45,2	85	33,7	**	197	70,4	52	55,3	**	463	75,2	241	57,7	***
	Not suitable for confinement	73	54,1	51	67,1		136	54,8	167	66,3		83	29,6	42	44,7		153	24,8	177	42,3	
**Household work**	Other persons/equally	125	92,6	64	86,5		178	72,1	166	66,1		N/I	N/I	N/I	N/I		N/I	N/I	N/I	N/I	
	Mostly by myself	10	7,4	10	13,5		69	27,9	85	33,9											
**Concern about living with household members**	Nothing or little	53	40,2	20	26,3	**	99	40,4	64	25,6	***	219	78,2	43	45,7	***	478	77,6	231	55,4	***
	Moderate, quite a bit or a lot	79	59,8	56	73,7		146	59,6	186	74,4		61	21,8	51	54,3		138	22,4	186	44,6	
**Concern about schooling**	Nothing or little	21	16,0	15	21,1		61	25,4	41	16,8		109	41,9	27	30,7		307	52,4	167	42,1	**
	No minors	29	22,1	14	19,7		63	26,3	39	16,0	***	10	3,9	4	4,5		23	3,9	17	4,3	
	Moderate, quite a bit or a lot	81	81,8	42	59,2		116	48,3	164	67,2		141	54,2	57	64,8		256	43,7	213	43,6	

*MH* Mental health

*N/I* No Information

* *p*-value < 0,001 in the comparison between men and women (chi2 test)

** *p*-value < 0,05 in the comparison between having or not having mental health problems in each sex (chi2 test)

*** *p*-value < 0,001 in the comparison between having or not having mental health problems in each sex (chi2 test)

### Mental health problems

Table 3 illustrates the correlations between the presence of mental health problems and the study variables. Regarding level of education, completion of university studies was associated with a protective factor against mental health problems in women from Brazil (aOR: 0.74, CI = 0.64;0.86), Peru (aOR: 0.57, CI = 0.33;0.99) and Spain (aOR: 0.50, CI = 0.36;0.70). With regard to age, younger age was associated with an elevated risk of mental health problems, with this association being particularly pronounced in the 18–34 years age group in Brazil [aOR men: 6.45 (CI = 3.44;12.07), aOR women: 3.56 (CI = 2.56;4.95)], Chile [aOR men: 23.95 (CI = 7.92;72.47), aOR women: 2.33 (CI = 1.26;4.31)] and Mexico [aOR men: 4.95 (CI = 2.01;12.19)].

**Table 3:** Association between having mental health problems and variables related to social and material factors of participants by sex in Brazil, Chile, Ecuador, Mexico, Peru and Spain in the first wave of COVID-19

		**Brazil**								**Chile**									**Ecuador**							
		**Men**			**Women**					**Men**				**Women**					**Men **					**Women**		
		*n*=1094			*n*=4138					*n*=963				*n*=1701					*n*=454					*n*=1015		
		aOR	IC 95%	*p*	aOR			IC 95%	*p*	aOR	IC 95%		*p*	aOR		IC 95%		*p*	aOR	IC 95%	*p*			aOR	IC 95%	*p*
**Educational level**	Nonuniversity studies	1,00			1,00				**	1,00				1,00					1,00					1,00		
	University studies	0,91	(0.68;1.23)		0,74			(0.64;0.86)		0,80	(0.59;1.10)			0,86		(0.67;1.11)			1,16	(0.63;2.17)				0,74	(0.50;1.10)	
**Age**	≥65	1,00		**	1,00				**	1,00			**	1,00				**	1,00					1,00		
	35-64	2,20	(1.19;4.08)		1,55			(1.14;2.13)		11,29	(3.69;34.5)			0,95		(0.52;1.73)			0,76	(0.22;2.65)				0,98	(0.17;5.57)	
	18-34	6,45	(3.44;12.07)		3,56			(2.56;4.95)		23,95	(7.92;72.47)			2,33		(1.26;4.31)			1,00	(0.29;3.43)				1,22	(0.22;6.90)	
**Pre-pandemic employment status**	Employed	1,00		*	1,00				*	1,00				1,00					1,00					1,00		
	Unemployed	1,46	(1.05;2.02)		1,20			(1.04;1.38)		1,08	(0.59;1.97)			0.89		(0.66;1.22)			1,04	(0.56;1.93)				1,29	(0.91;1.82)	
**Change in employment status during the pandemic**	No change/improvement	1,00		**	1,00				**	1,00			*	1,00				**	1,00		*			1,00		*
	Worsened	2,41	(1.81;3.21)		1,64			(1.43;1.87)		1,45	(1.09;1.92)			1,49		(1.2;1.86)			1,81	(1.17;2.80)				1,52	(1.13;2.03)	
**Housing tenure**	Own home	1,00		**	1,00				**	1,00				1,00				*	1,00					1,00		
	Lease or rent	1,65	(1.20;2.28)		1,26			(1.07;1.48)		1,08	(0.78;1.48)			1,41		(1.09;1.82)			1,49	(0.92;2.42)				1,24	(0.89;1.73)	
	Living in someone else´s home	2,34	(1.31;4.15)		1,38			(1.06;1.79)		1,01	(0.63;1.62)			1,51		(1.05;2.15)			1,21	(0.60;2.43)				1,30	(0.85;2.00)	
**Perception of adequate housing**	Suitable for confinement	1,00		*	1,00				**	1,00			*	1,00				**	1,00					1,00		*
	Not suitable for confinement	1,40	(1.06;1.85)		1,56			(1.37;1.79)		1,51	(1.13;2.03)			1,73		(1.35;2.21)			1,02	(0.68;1.54)				1,42	(1.08;1.88)	
**Household work**	Other persons/equally among household members	1,00			1,00				**	1,00			*	1,00				*	1,00					1,00		*
	Mostly by myself	1,26	(0.89;1.79)		1,30			(1.13;1.48)		1,97	(1.32;2.94)			1,30		(1.04;1.62)			1,38	(0.74;2.56)				1,65	(1.24;2.18)	
**Concern about living with household members**	Nothing or little	1,00		**	1,00				**	1,00			**	1,00				**	1,00		*			1,00		**
	Moderate, quite a bit or a lot	1,93	(1.4;2.67)		1,59			(1.38;1.84)		2,10	(1.57;2.82)			2,10		(1.68;2.61)			2,01	(1.33;3.04)				2,16	(1.64;2.84)	
**Concern about schooling**	Nothing or little	1,00			1,00					1,00				1,00					1,00					1,00		
	No minors	1,27	(0.77;2.11)		1,15			(0.93;1.42)		1,24	(0.82;1.88)			1,16		(0.86;1.57)			1,67	(0.76;3.66)				1,15	(0.69;1.91)	
	Moderate, quite a bit or a lot	1,22	(0.73;2.04)		1,17			(0.95;1.44)		0,86	(0.61;1.22)			1,07		(0.84;1.38)			1,35	(0.66;2.79)				1,08	(0.67;1.73)	
		**México**								**Perú**									**España**							
		**Men**				**Women**				**Men **					**Women**				**Men**				**Women**			
		*n*=1311				*n*=3268				*n*=184					*n*=404				*n*=347				*n*=977			
		aOR	IC 95%	*p*		aOR	IC 95%		*p*	aOR	IC 95%	*p*			aOR	IC 95%	*p*		aOR	IC 95%		*p*	aOR		IC 95%	*p*
**Educational level**	Nonuniversity studies	1,00				1,00				1,00					1,00		*		1,00				1,00			**
	University studies	0,81	(0.61;1.08)			0,90	(0.75;1.06)			0,74	(0.32;1.69)				0,57	(0.33;0.99)			1,27	(0.69;2.35)			0,50		(0.36;0.70)	
**Age**	≥65	1,00		**		1,00			**	1,00					1,00				1,00			*	1,00			
	35-64	3,43	(1.39;8.46)			1,01	(0.56;1.82)			0,49	(0.04;6.19)				0,93	(0.23;3.77)			1,09	(0.11;10.37)			3,43		(0.39;29.94)	
	18-34	4,95	(2.01;12.19)			1,78	(0.99;3.20)			1,06	(0.08;13.36)				1,55	(0.37;6.42)			3,32	(0.33;33.18)			4,94		(0.55;44.35)	
**Pre-pandemic employment status**	Employed	1,00				1,00				1,00					1,00				1,00				1,00			
	Unemployed	1,06	(0.77;1.44)			0,90	(0.76;1.07)			1,04	(0.32;3.40)				1,03	(0.54;1.96)			1,28	(0.55;2.97)			0,70		(0.48;1.00)	
**Change in employment status during the pandemic**	No change/improvement	1,00		**		1,00			**	1,00		*			1,00				1,00				1,00			*
	Worsened	2,03	(1.59;2.58)			1,61	(1.39;1.86)			2,11	(1.03;4.32)				1,35	(0.88;2.09)			1,51	(0.88;2.58)			1,51		(1.15;1.99)	
**Housing tenure**	Own home	1,00				1,00			*	1,00					1,00				1,00				1,00			*
	Lease or rent	1,23	(0.89;1.70)			1,09	(0.89;1.33)			1,36	(0.63;2.91)				1,10	(0.64;1.89)			0,85	(0.45;1.58)			0,71		(0.50;1.01)	
	Living in someone else´s home	1,28	(0.92;1.79)			1,28	(1.06;1.54)			2,74	(0.98;7.68)				0,96	(0.53;1.74)			1,28	(0.28;5.86)			0,43		(0.17;1.12)	
**Perception of adequate housing**	Suitable for confinement	1,00				1,00			*	1,00					1,00				1,00				1,00			**
	Not suitable for confinement	1,21	(0.94;1.55)			1,20	(1.03;1.40)			1,36	(0.64;2.90)				1,25	(0.80;1.95)			1,34	(0.77;2.34)			1,74		(1.30;2.34)	
**Household work**	Other persons/equally among household members	1,00				1,00			**	1,00					1,00					N/I					N/I	
	Mostly by myself	1,10	(0.77;1.58)			1,51	(1.29;1.76)			1,81	(0.60;5.48)				1,17	(0.74;1.86)										
**Concern about living with household members**	Nothing or little	1,00		**		1,00			**	1,00		*			1,00		*		1,00			**	1,00			**
	Moderate, quite a bit or a lot	2,52	(1.96;3.25)			1,83	(1.58;2.12)			2,32	(1.05;5.10)				1,73	(1.08;2.76)			4,31	(2.47;7.51)			2,36		(1.75;3.19)	
**Concern about schooling**	Nothing or little	1,00		**		1,00				1,00					1,00		*		1,00				1,00			
	No minors	1,12	(0.77;1.61)			1,02	(0.82;1.27)			0,52	(0.17;1.59)				1,08	(0.56;2.10)			1,62	(0.41;6.42)			0,90		(0.44;1.82)	
	Moderate, quite a bit or a lot	0,63	(0.46;0.88)			0,97	(0.80;1.18)			0,44	(0.17;1.18)				2,18	(1.25;3.82)			1,24	(0.69;2.22)			1,29		(0.97;1.72)	

*aOR* Adjusted Odd Ratio

*N/I* No Information

* *p* < 0,05

** *p* < 0,001

With respect to changes in working conditions, an association was observed between worsening work and mental health problems, particularly among men in Brazil [aOR: 2.41 (CI = 1.81;3.21)] and Mexico [aOR Men: 2.03 (CI = 1.59;2.58)]. In Chile, the associations were similar between men and women (aOR men: 1.45, CI = 1.09;1.92; aOR women: 1.49, CI = 1.20;1.86), Ecuador (aOR men: 1.81, CI = 1.17;2.80; aOR women: 1.52, CI = 1.13;2.03), and men in Peru (aORa: 2.11, CI = 1.03;4.32). In Spain, the association was significant only for women (ORa: 1.51, 95% CI: 1.15, 1.99). Among individuals who were unemployed prior to the onset of syndemic conditions, an association with mental health issues was identified in Brazil [aOR men: 1.46 (CI = 1.05;2.02); aOR women: 1.20 (CI = 1.04;1.38)].

With regard to housing tenure, an association was identified between renting or residing in another person’s home and the prevalence of mental health problems. For individuals residing in another person’s home in Brazil, the odds ratio (OR) for men was 2.34 (95% confidence interval (CI): 1.31, 4.15), while for women it was 1.38 (95% CI: 1.06, 1.82). Furthermore, an association was observed in women from Chile (ORa: 1.51 (CI = 1.05;2.15)) and Mexico (aOR = 1.28 (CI = 1.06;1.54)). With respect to poor perceptions of housing conditions, an association was observed in Brazil [aOR men: 1.40 (CI = 1.06; 1.85) aOR women: 1.56 (CI = 1.37; 1.79)], Chile [aOR men: 1.51 (CI = 1.13; 2.03) aOR women: 1.73 (CI = 1.35; 2.21)], women from Ecuador [aOR = 1.42 (CI = 1.08; 1.88)], Mexico [aOR = 1.20 (CI = 1.03; 1.40)], and Spain [aOR = 1.74 (CI = 1.30; 2.34)].

Among individuals who reported engaging in caregiving activities, the presence of mental health problems was associated with Brazil (aOR women: 1.30; CI = 1.13;1.48) and Chile (aOR men: 1.97; CI = 1.32;2.94), as well as women in Ecuador (aOR = 1.65) and Mexico (aOR = 1.51). Additionally, the data revealed that women in Ecuador [aOR = 1.65 (CI = 1.24;2.18)] and Mexico [aOR = 1.51 (CI = 1.29;1.76)] exhibited a similar trend. A correlation was identified between concern about living with others in the household and mental health problems in both men and women across all countries, with a stronger association observed in men from Spain [aOR: 4.31 (CI = 2.47;7.51)], women from Spain [aOR: 2.36 (CI = 1.75;3.19)], and men from Mexico [aOR: 2.52 (CI=(1.96;3.25)]. Concern about children’s schooling in the household was associated with mental health problems in women from Peru (aOR: 2.18, CI = 1.25;3.82), whereas it was a protective factor in men from Mexico (aOR: 0.63, CI = 0.46;0.88).

## Discussion

The present study examined the effects of sociostructural factors on the mental health risks of individuals residing in Brazil, Chile, Ecuador, Mexico, Peru, and Spain who were responsible for the care of minors or dependents during the initial phase of the global pandemic, specifically the period of lockdown. One of the most notable findings is the markedly higher prevalence of women with minor children or dependents experiencing mental health issues across all countries studied, with a comparatively lower incidence observed in Spain. Among the associated risk factors were poor housing conditions and a university education, while having a university education was found to be a protective factor. With regard to men, the risk was observed to be associated with unfavorable working conditions and concerns about cohabiting at home.

For those assuming UCW, there was a notable tendency to report mental health problems, particularly among women in all countries (with the exception of Peru and Spain), underscoring the heightened pressure they faced during the lockdown. The causes of poor mental health are numerous, but during the period of lockdown, they were linked to various factors, including isolation, a lack of resources (monetary, material, and so forth), poor social networks, and a deficiency of support at the state level ( [28]. Furthermore, women who assumed the UCW role were required to fulfil multiple additional responsibilities, such as the roles of educator and counsellor, without any breaks or respite from these duties [29]. The burden of multiple roles may result in elevated mental stress and burden, which can impact mental health [30]. In this regard, the prolonged exposure to stressful circumstances during the lockdown period, coupled with the heightened sense of job insecurity and the additional responsibilities associated with healthcare roles and the care of sick family members, also contributed to the deterioration of mental health [30].

The relationship between poor housing conditions and mental health problems has been identified as a significant concern, particularly among women. This emphasizes the necessity to consider structural factors such as living space and housing conditions [31]. The correlation between inadequate thermal insulation or high humidity and socio-economic status has been identified as a contributing factor to energy poverty and poor health outcomes [32]. In the context of syndemia, this was further compounded by the absence of guarantees regarding the security of housing tenure and the obligation to remain in the residence, which resulted in the indiscriminate assumption of productive and reproductive work and the inadequate allocation of spaces for each task. The quality of housing is also associated with socioeconomic indicators. Therefore, the right to decent and adequate housing is regarded as a fundamental necessity for individuals to engage in their daily activities seamlessly and harmoniously [33].

Another social factor indicated that university education was a protective factor for mental health, particularly among women from Brazil, Peru, and Spain. This finding is consistent with the existing literature, which suggests that a higher level of education may be associated with greater resources and economic stability, and therefore less uncertainty, especially when attempting to reconcile multiple roles within the household. It should also be noted that there may be more support from partners (who telework), other family members, or paid help, which could imply more care networks [34].

Conversely, among men who reported a deterioration in their employment circumstances, there was an elevated risk of mental health problems. In a context of socioeconomic crisis, poor working conditions have a destabilizing effect on the caregiving relationship from a material perspective. This places strain on the breadwinner model, increasing the anxiety of having to respond to the needs and demands of the family [35]. In a model where traditional roles are reproduced, the lack (or scarcity) of family income can have a detrimental impact on the health of men, who have historically been expected to act as the main *breadwinner* [36]. In light of these findings, it becomes evident that there is an urgent need for economic and mental health support policies for those affected by worsening labor market conditions. Furthermore, the long-term paradigm shifts are needed regarding the potential asymmetry of economic power within families with a particular focus on the feminization of poverty.

The findings of our study indicate that concern about cohabitation had a more pronounced impact on men. This may be attributed to the absence of established practices for managing productive and reproductive work (such as parenthood) within a framework of obligatory coexistence [30]. The implication of care work for men may affect them in that they have smaller and less diverse support networks, along with a tendency not to share their feelings with friends, family, and health professionals [37]. This underscores the importance of emotional management and social organization in situations where individuals are required to share confined spaces for extended periods with those who are dependent on them. However, another study observed that men were able to perceive positive coexistence and management of domestic work during the lockdown period. This may be attributed to their perception that the primary responsibility did not fall on them [38].

Additionally, the findings revealed discrepancies across the countries under examination, with a notable reduction in the likelihood of mental health problems in Spain. This is an intriguing finding, given that Spain, within the European context, is one of the countries that allocates the fewest resources to care policies. However, when compared with Latin American countries, the situation is reversed [17]. The implementation of care policies that address gender issues and are grounded in human rights can also play a pivotal role in transforming the traditional sexual division of labor in households, thereby facilitating a shift in perceptions regarding care responsibilities. For instance, a positive correlation has been identified between the introduction of parental leave at the national level and an increase in the amount of time spent caring for children by men [39]. Furthermore, research suggests that policies that facilitate women’s labor participation and mitigate their caregiving responsibilities, such as public services, family support and parental rights, are associated with a reduction in gender-based health disparities [20].

Despite recent efforts to improve the circumstances of caregivers in Latin America, the onset of the SARS-CoV-2 pandemic has exacerbated disparities in the quality of life of those in caregiving roles [40]. The global pandemic has served to exacerbate existing inequities and introduce novel and more far-reaching challenges, many of which are linked to the expansion of social protection systems in countries around the world [41]^,^. It is crucial to promote gender equity and challenge the traditional sexual division of roles, advocating for co-responsibility [40]. Furthermore, it is essential to strengthen governmental assistance to caregivers through the implementation of support programs and to assign greater value to their work [42]. Furthermore, it is imperative to ascribe social and symbolic value to the role of caregiving, given its pivotal role in maintaining societal life.

The principal limitation of this study was the utilization of an online survey, which may have resulted in the exclusion of individuals lacking access to technology or those with limited digital literacy. Another limitation was the discrepancy in the size of the samples collected in each country. It is therefore recommended that the results be interpreted with caution. Although this is a sample of people who live with dependents or minors, it would have been preferable to focus the sample on people who reported care work. However, the decision was taken to make this distinction in order to include Spain within the countries (a question not included in that country). This study has several notable strengths. It is one of the first to examine the impact of the lockdown on this group in Latin American countries and Spain. This allows for a comprehensive understanding of the experiences of this population during the initial phase of the syndemic and mental health crisis. The study’s focus on a feminist perspective is particularly timely and important in this context.

## Conclusion

In conclusion, the period of lockdown presented a significant challenge for individuals living with dependents or minors, resulting in adverse effects on their mental health. The most affected group was women, particularly those with lower levels of education, residing in less favorable housing conditions and assuming the primary caregiving role. For men, these factors were associated with deteriorating working conditions and concerns about cohabitation. Moreover, disparities were observed across the countries examined, with reduced mental health risks among individuals from Spain, highlighting the significance of social protection systems and sociostructural determinants. It is imperative that multidimensional and intersectoral strategies for the protection and support of carers are developed, promoting the formation of robust support networks and the provision of adequate spaces, and advocating for an equitable distribution of these responsibilities. In particular, the well-being of caregivers has an impact on the well-being of those they care for, as well as future generations.

## Data Availability

The study database is available upon reasonable request, following approval of a proposal and with signed data-access agreement.

## Abbreviations

COVID-19: Coronavirus Disease 2019
UCW: Unpaid Care Work
GAD-7: Generalized Anxiety Disorder Scale
PHQ-9: Patient Health Questionnaire
aORs: Adjusted odds ratios
MH: Mental health
IDIAPJGol: Institut de Recerca en Atenció Primària Jordi Gol i Gurina
